# Cannabidiol as a treatment for epilepsy

**DOI:** 10.1007/s00415-017-8663-0

**Published:** 2017-11-09

**Authors:** William O. Pickrell, Neil P. Robertson

**Affiliations:** 1 Neurology and Molecular Neuroscience, Swansea University Medical School, Swansea University, Swansea, SA2 8PP UK; 2 Institute of Psychological Medicine and Clinical Neurosciences, Cardiff University, Cardiff, CF14 4XW UK

Despite an increasing number of anti-epileptic drugs (AEDs), the proportion of drug-resistant cases of epilepsy has remained fairly static at around 30% and the search for new and improved AEDs continues. Cannabis has been used as a medical treatment for epilepsy for thousands of years; it contains many active compounds, the most important being tetrahydrocannabinol, which has psychoactive properties, and cannabidiol, which does not. Animal models and clinical data to date have suggested that cannabidiol is more useful in treating epilepsy; there is limited evidence that tetrahydrocannabinol has some pro-convulsant effects in animal models. The mechanism by which cannabidiol exerts its anti-convulsant properties is currently unclear.

This month’s journal club reviews three papers using cannabidiol for the treatment of epilepsy. The first paper describes a small case series where cannabidiol is used in a rare pediatric epilepsy syndrome, febrile infection-related epilepsy syndrome (FIRES); the second paper is a larger open label study of cannabidiol in a variety of refractory epilepsies and the third paper describes a randomized control trial of cannabidiol in Dravet syndrome.

## Cannabidiol as a potential treatment for febrile infection-related epilepsy syndrome (FIRES) in the acute and chronic phases

This study is a case series of seven children from five US hospitals. All of the children had the rare but devastating febrile infection-related epilepsy syndrome (FIRES). FIRES affect school age children and is characterized by a febrile illness followed by very frequent multi-focal seizures with refractory status epilepticus (acute phase). The chronic phase consists of pharmacoresistant epilepsy with significant cognitive and behavioral effects. The etiology of FIRES is unknown and there is currently no effective treatment, although the ketogenic diet has shown some promise.

The children in this study had an extensive workup for other causes of new onset status epilepticus. They were given cannabidiol using an emergency or expanded access investigational new drug protocol. Two children started cannabidiol in the acute phase with the remainder starting the drug in the chronic phase. Before starting cannabidiol, the participants had trialed a median of seven anti-epileptic drugs, had been given steroids or intravenous immunoglobulin and all but one of the children had been given a ketogenic or modified Atkins diet. Five of the participants had trials of multiple anesthetic agents. One participant was given 15 mg/kg/day of cannabidiol, one was given 20 mg/kg/day with the remainder having the maximum dose of 25 mg/kg/day.

One of the two patients treated in the acute stage of FIRES died of multi-organ failure; the other had a cessation of their status epilepticus. In the remaining five patients treated in the chronic phase, there was a mean decrease in seizure frequency of 91% 4 weeks after and 65% 48 weeks after treatment when compared with pre-treatment seizure frequency.

*Comment*. This was a small case series in a very rare epilepsy syndrome and the usual caution needs to be exercised when interpreting these results. Despite this, the results seem to suggest that cannabidiol can be considered in FIRES as an additional therapy when conventional therapies have failed.

Gofshteyn JS et al (2017) J Child Neurol 32(1):35–40.

## Cannabidiol in patients with treatment-resistant epilepsy: an open-label interventional trial

This is an open-label study series of children and young adults across eleven US epilepsy centers. Inclusion criteria were that the patients were aged between 1 and 30 years, had intractable childhood onset epilepsy, had four or more seizures with a motor component per month, and were on stable doses of antiepileptic drugs for at least a month before enrolment. Patients were given cannabidiol and doses were titrated to either 25 or 50 mg/kg/day. The primary endpoints were safety and tolerability of cannabidiol (safety analysis group) and median change in mean seizure frequency at 12 weeks compared to baseline (efficacy analysis group). Parents and carers recorded seizure frequency using seizure diaries.

214 patients were recruited, 52 did not have 3 months of follow-up data, 11 dropped out before 12 weeks because of adverse effects, worsening seizures, or sudden death in epilepsy (1 patient). 162 patients were included in the safety analysis group. There were a variety of underlying severe epilepsies including Dravet syndrome (20%) and Lennox Gastaut syndrome (19%). A further 25 patients were excluded from the safety analysis group (mainly due to not having motor seizures) to leave 137 patients in the efficacy group.

Adverse events were reported in 80% of the safety group, these included somnolence (25%), decreased appetite (19%), diarrhea (19%), fatigue (13%), convulsions (11%), increased appetite (9%) and status epilepticus (8%). Serious adverse events were reported in 48 (30%) patients, including one death—a sudden unexpected death in epilepsy regarded as unrelated to study drug. Of these, 20 (12%) patients had severe adverse events thought to be possibly related to cannabidiol use. Of the 13 cases of status epilepticus, nine were thought to be possibly related to cannabidiol use although none of these were associated with reductions in cannabidiol or other AEDs.

There was a reduction of 36.5% in the median monthly motor seizure frequency when compared to baseline. 39% of patients had a reduction by more than 50% in their motor seizures and 21% had a reduction by more than 70% in their motor seizure frequency.

*Comment.* This is an open-label trial and caution must be exercised in interpreting the results without a control group. This study did demonstrate a side effect and efficacy profile for cannabidiol which certainly compares with the AEDs that would be considered in the group of patients studied in this trial and it would seem to suggest that cannabidiol is safe to use in this group in the short term.

Devinsky O et al (2016) Lancet Neurol 15(3):270–278.

## Trial of cannabidiol for drug-resistant seizures in the Dravet syndrome

This was a double-blind, randomized, placebo controlled trial of cannabidiol in children and young adults with Dravet syndrome. Dravet syndrome is a severe epileptic encephalopathy with onset typically in the first year of life. Initially seizures are fever related, other seizures types follow, including generalized tonic–clonic seizures, myoclonic seizures and atypical absences, which are typically refractory to treatment. Most children with Dravet syndrome have a significant degree of intellectual disability. 75% of cases have mutations in the sodium channel gene *SCN1A*.

Patients were recruited across 23 centers in the USA and Europe and were eligible if they had a diagnosis of Dravet syndrome, were taking at least one AED, and had at least four convulsive seizures in a baseline (4-week) period. After the baseline period, there were periods of treatment (14 weeks), treatment tapering (10 days) and safety follow-up (4 weeks). The primary end point was a change in monthly convulsive seizure frequency. Seizures were recorded daily by patients or their carers using a voice activated recording system. There were also secondary end points which included categorical measures of improvement, seizure duration, and behavior as well as changes in different seizure types.

120 patients were randomized (61 cannabidiol and 59 placebo) and 108 patients completed the trial (52 in the cannabidiol group and 56 in the placebo group). Nine patients withdrew from the cannabidiol group (eight because of adverse effects) and three patients were withdrawn from the placebo group (one because of an adverse event). The mean age of the participants was 9.8 years; they had a baseline median monthly seizure frequency of 120 seizures per month and were currently taking a mean of 2.9 AEDs having previously tried a mean of 4.6 AEDs.

The median monthly convulsive seizure frequency decreased by 38.9% in the cannabidiol group and by 13.3% in the placebo group. This was a statistically significant difference, *p* = 0.01. There was a similar difference between the groups in terms of total number of seizures with the cannabidiol group having a reduction in monthly median total seizure frequency of 28.6% and the placebo group having a reduction in monthly medial total seizure frequency of 9%, *p* = 0.03 for a difference between the groups. There was not a statistically significant difference between the groups in terms of the numbers of patients who had at least a 50% reduction in seizure frequency (43% in the cannabidiol group versus 27% in the placebo group, *p* = 0.08).

75% of the patients in the cannabidiol group were deemed to have adverse effects associated with the drug compared with 36% of the patients in the placebo group. Common adverse effects in the cannabidiol group included fatigue/somnolence, diarrhea, decreased appetite, vomiting and pyrexia. Twelve patients in the cannabidiol group had a raised liver aminotransferase level compared with one patient in the placebo group.

*Comment*. This is a significant paper given that it describes a randomized control trial of cannabidiol in epilepsy. It showed that cannabidiol resulted in a greater reduction in convulsive-seizure frequency than placebo in patients with drug-resistant Dravet syndrome and would suggest that cannabidiol is a real treatment option in this patient group. The results need to be taken in the context that there were some adverse drug effects and Dravet’s syndrome is rare and genetically homogeneous, and so this approach might not be immediately transferable to more common epilepsies.

Devinsky O et al (2017) N Engl J Med. 377(7):699–700.

*Conclusion.* The evidence is increasing that cannabidiol is an effective treatment option for childhood onset severe treatment-resistant epilepsies with a tolerable side effect and safety profile. Further evidence is needed before cannabidiol can be considered in more common or adult onset epilepsies. Longer-term safety data for cannabidiol, particularly considering its effects on the developing brain, are also required.

